# Resveratrol accelerates wound healing by inducing M2 macrophage polarisation in diabetic mice

**DOI:** 10.1080/13880209.2022.2149821

**Published:** 2022-12-05

**Authors:** Youjun Ding, Ping Yang, Shiyan Li, Hao Zhang, Xiaofeng Ding, Qian Tan

**Affiliations:** a Department of Burns and Plastic Surgery, Nanjing Drum Tower Hospital Clinical College of Jiangsu University, Nanjing, China; b Department of Emergency Surgery, The Fourth Affiliated Hospital of Jiangsu University (Zhenjiang Fourth People’s Hospital), Zhenjiang, China; c Department of Burns and Plastic Surgery, Nanjing Drum Tower Hospital, the Affiliated Hospital of Nanjing University Medical School, Nanjing, China; d Department of Burns and Plastic Surgery, Nanjing Drum Tower Hospital Clinical College of Traditional Chinese and Western Medicine, Nanjing University of Chinese Medicine, Nanjing, China; e Department of Burns and Plastic Surgery, Anqing Shihua Hospital of Nanjing Drum Tower Hospital Group, Anqing, China

**Keywords:** Diabetic wounds, inflammation, STZ, THP-1, PI3K/Akt

## Abstract

**Context:**

The reduction in M2 macrophage polarisation plays a major role during diabetic wound healing. Resveratrol (RSV) can promote the polarisation of M2 macrophages and accelerate diabetic wound healing. However, the specific mechanism by which RSV regulates M2 macrophage polarisation to promote diabetic wound healing is unclear.

**Objective:**

This study evaluated the effectiveness of RSV on diabetic wound healing and analysed the underlying mechanisms.

**Materials and methods:**

STZ-induced C57/B6 mice were used as a diabetic mice model for a period of 15 days. RSV (10 μmol/L) was injected around the wound to evaluate the effect of RSV on the healing process of diabetic wounds. The human monocyte line THP-1 was used to evaluate the effects of RSV (10 μmol/L) on polarisation of M2 macrophages and the secretion of pro-inflammatory factors.

**Results:**

*In vivo*, RSV significantly increased diabetic wound healing (*p* < 0.05) and make the regenerated skin structure more complete. And it promoted the expression of α-SMA and Collagen I (*p* < 0.05). Moreover, RSV reduced the secretion of inflammatory factors (TNF-α, iNOS and IL-1β) (*p* < 0.05) and promoted M2 macrophage polarisation by increasing Arg-1 and CD206 expression (*p* < 0.01). *In vitro*, RSV promoted the polarisation of M2 macrophages (*p* < 0.001) and reduced the secretion of pro-inflammatory factors (TNF-α, IL-6 and IL-1β) (*p* < 0.05). The therapeutic effects of RSV were all significantly reversed with LY294002 (*p* < 0.01).

**Discussion and conclusions:**

RSV has the positive effects on promoting the acceleration and quality of skin wound healing, which provides a scientific basis for clinical treatment in diabetic wound.

## Introduction

Diabetes mellitus is one of the most common chronic metabolic diseases (Zheng et al. 2018). Since 2019, 463 million people have been diagnosed with diabetes, and its prevalence rate is increasing globally (Saeedi et al. 2019). Patients with diabetes usually develop severe complications owing to a chronic hyperglycaemic environment. Diabetic foot ulcer (DFU) is one of the most common and devastating complications, affecting at least 15% of diabetic patients, among whom approximately 20% experience non-traumatic amputations and even death (Armstrong et al. 2017; Monteiro-Soares et al. 2019). DFU imposes a heavy financial burden on patients and society (Bellary et al. 2021). However, the mechanism of DFU remains unclear, and effective treatments are urgently needed.

Normal wound healing involves four dynamic and overlapping stages: hemostasis, inflammation, proliferation, and remodelling (Eming et al. 2007; Patel et al. 2019). However, diabetic wounds often stagnate during the inflammatory phase due to persistent chronic inflammation (Dardmah and Farahpour 2021). It has been shown that wound healing can be accelerated, and the inflammatory phase curtailed by promoting wound cell proliferation (Daemi et al. 2019; GHaraboghaz et al. 2020). Pro-inflammatory macrophages (M1) fail to polarise to anti-inflammatory macrophages (M2) in a timely manner and are the leading cause of unresolved inflammation in DFU (Wolf et al. 2021). Under normal conditions, M1 macrophages promote the transient inflammatory response in the early inflammation stage and clear necrotic tissue debris and pathogens around the wound by secreting high levels of pro-inflammatory factors (IL-6, IL-1β, and TNF-α), reactive oxygen species, and proteases (Aitcheson et al. 2021). Macrophages are then polarised to M2 and secrete growth factors (vascular endothelial growth factor (VEGF), fibroblast growth factor (FGF), and epidermal growth factor (EGF)) and anti-inflammatory cytokines (IL-10, and IL-4) to regulate the proliferation and differentiation of cells around the wound during the late inflammatory response (Wynn and Vannella 2016; Shen et al. 2020). In contrast, M1 macrophages cannot polarise to M2 macrophages in diabetic wounds promptly, which results in a prolonged and sustained inflammatory state, suppressing angiogenesis and collagen deposition, ultimately leading to delayed or even non-healing wounds (Falanga 2005; Boniakowski et al. 2017; Komi et al. 2020). Previous studies have demonstrated that improving macrophage phenotypic switching could protect against excessive inflammation and accelerate diabetic wound healing (He et al. 2017; Hu et al. 2020; Li et al. 2022). Accordingly, promoting M2 polarisation of macrophages is an effective treatment for diabetic wounds.

Resveratrol (RSV) is a novel regulator of inflammation and M2 macrophage polarisation in macrophages. A previous study showed that RSV promotes BV-2 mouse microglial polarisation to the M2 phenotype and reduces lipopolysaccharide (LPS)-induced neuroinflammation (Wang et al. 2020). It has also been shown that RSV delivered by tetrahedral framework nucleic acid nanoparticles could switch M1 phenotype macrophages to M2 phenotype macrophages in several tissues (Li et al. 2021). These studies suggest that RSV can upregulate the expression of M2 subtypes (Arg-1, CD206, and IL-10) and decrease the expression of M1 subtypes (IL-6, IL-1β, iNOS, and CD86). Phosphoinositide 3-kinase/protein kinase B (PI3K/Akt) signalling is an important pathway that modulates macrophage polarisation (Linton et al. 2019). Previous studies have demonstrated that RSV attenuates the inflammatory response and related tissue injury *via* the PI3K/Akt pathway (Radwan and Karam 2020; Frontiers Production Office 2021). In addition, recent studies have revealed that RSV has positive effects on both normal and diabetic wound healing (Huang et al. 2019; Kaleci and Koyuturk 2020; Pignet et al. 2021). However, the effects of RSV on macrophage polarisation in diabetic wounds remain unclear.

Our study is the first to investigate the specific mechanisms by which RSV promotes wound healing and affects M2 macrophage polarisation in diabetic mice, using RSV regulation of macrophage polarisation *via* the PI3K/Akt pathway as an entry point.

## Materials and methods

### Materials

Glucose (D9434), RSV, streptozotocin (STZ; S0130), phorbol 12-myristate 13-acetate (PMA; P8139), dimethyl sulphoxide (DMSO; D2650), and LPS (L2654) were obtained from Sigma-Aldrich (France). The PE anti-human CD206 (321106) antibody and the FITC anti-human CD68 (333806) antibody were bought from BioLegend. Primary antibodies against Arginase 1 (Arg-1; 93668), α-SMA (19245S), p-AKT (13038S), and AKT (9272S) were provided by Cell Signal Technology (CST). Collagen I (ab260043), IL-1β (ab254360), CD206 (ab64693), TNF-α (ab205587), and GAPDH (ab8345) were obtained from Abcam. The PI3K/AKT pathway inhibitor, LY294002, was obtained from Selleckchem. Trizol Reagent and SYBR Green dye were obtained from Vazyme. RSV was dissolved in DMSO to 1 mM and stored at −20 °C.

### Animal model construction

All animal experiments were approved by the Animal Ethics Committee of the First Nanjing Hospital affiliated with Nanjing Medical University (DWSY-21059103). Male C57/B6 mice (weighing 19–23 g, 6–8 weeks old) were purchased from Jiangsu Jicui Yaokang Biotechnology and maintained in a 12 h light/dark cycle of under specific conditions. After 1 week of adaptive feeding, mice were randomly assigned to three groups: the control (NC), diabetes (DM), and diabetes + RSV (DR) groups (16 mice per group). Diabetes was induced in mice by intraperitoneal injection of STZ (50 mg/kg), which was freshly prepared in sodium citrate buffer solution (pH = 4.5). Mice with a blood glucose level > 16.7 mmol/L over 1 month were considered to have diabetes. Then, a 1 cm diameter full-thickness excisional wound was created on the dorsal side of the mice. For the DR group, 100 µL RSV (10 μmol/L) was injected intradermally around the wound on days 0, 1, 2, 3, 5, 7, 9, and 11 following injury; the mice in the NC and DM groups were injected around the wound with an equal volume PBS (100 µL) as a control treatment. Images of the wound areas were taken on days 0, 3, 7, 10, 13, and 15 post-operation and analysed using ImageJ software (National Institutes of Health). The skin around the wound was collected on days 10 and 15 for subsequent experiments.

### Cell culture and treatment

Human monocyte cell line THP-1 was offered by the Cell Bank of the Chinese Academy of Sciences and cultured in RPMI1640 (Gibco, USA) containing 33 mM _D_-glucose and 10% foetal bovine serum (Gibco, USA). To induce the differentiation of THP-1, the cells were stimulated with PMA (100 nmol/L) for 24 h, then followed by 48 h of exposure to LPS (100 ng/mL). After 72 h of induction, RSV (10 μmol/L) or an equal volume of PBS was added and incubated for 48 h. Furthermore, a specific PI3K/Akt pathway inhibitor was used before RSV treatment: 25 μmol/L LY294002 was pre-treated for 24 h.

### CCK‑8 cell viability assay

The activity of THP-1 cells at different RSV concentrations was determined using the CCK-8 assay. THP-1 cells were inoculated into 96-well plates and then treated with RSV at concentrations of 0, 5, 10, 20, 40, 80, and 160 μmol/L for 24 h, where concentration 0 was used as the control group. THP-1 cells were then incubated with CCK-8 working solution for 2 h at 37 °C, and then the absorbance at 450 nm was measured by spectrophotometer (Thermo, USA) (Yang et al. 2014). Cell viability was calculated by the absorbance ratio of each treatment group to the control group.

### Flow cytometry

To identify the percentage of M2 macrophages in total macrophages, PE-conjugated anti-CD206 monoclonal antibody and FITC conjugated anti-CD68 monoclonal antibody was used to preincubate the cells and then stained in the dark at 4 °C for 30 min. The samples were analysed using a FACS Calibur Aria^TM^ II flow cytometer. The data were analysed using FlowJo 10.0 software.

### Western blotting analysis

A total Protein Extraction Kit (Solarbio, Beijing) was used for protein extraction from wound tissues and macrophages, and the protein concentration was determined using a BCA assay kit (Solarbio, Beijing). After separation on a 10% SDS-PAGE gel, the proteins were blotted onto a polyvinylidene fluoride membrane (Bio-Rad Inc., USA). The membrane was blocked with TBST buffer containing 5% bovine serum albumin (BSA). After blocking, the membrane was incubated with the corresponding primary antibody at 4 °C overnight and incubated with the secondary antibody at room temperature for 1 h. Finally, all blots were investigated using an enhanced chemiluminescence reagent (Vazyme, China) and recorded using the Tanon luminescence imaging system.

### RNA purification and RT-qPCR analysis

Trizol reagent was used to extract total RNA from the skin tissues and macrophages. Reverse transcription of the total RNA was performed using HiScript III-RT Reagent Kit (Vazyme, China). SYBR Green detection reagent was used for RT-qPCR analysis. After GAPDH normalisation, the target gene relative expression levels were determined using the 2^−ΔΔ^*^CT^* approach. The primer sequences used are displayed in Table 1.

**Table 1.** Primer sequence used for RT-qPCR.

Primer name	Forward primers	Reverse primers
Human-Arg-1	ACTTAAAGAACAAGAGTGTGATGTG	CATGGCCAGAGATGCTTCCA
Human-CD206	AATGCTACCACAGTTATGCCTAC	TTCGTGCCTCTTGCCAATT
Human-IL-1β	TGATGGCTTATTACAGTGGCAA	TAGTGGTGGTCGGAGATTCG
Human-IL-6	AGTGAGGAACAAGCCAGAGC	GGTCAGGGGTGGTTATTGCA
Human-GAPDH	GAGTCAACGGATTTGGTCGT	GACAAGCTTCCCGTTCTCAG
Mouse-Arg-1	CTCCAAGCCAAAGTCCTTAGAG	AGGAGCTGTCATTAGGGACATC
Mouse-D206	AGCAGATGGAAGGTCTATGGAA	TGTCGTAGTCAGTGGTGGTT
Mouse-TNF-α	CGTGGAACTGGCAGAAGAG	TGAGAAGAGGCTGAGACATAGG
Mouse-IL-1β	TTCCTGAACTCAACTGTGAAATGC	TGTTGATGTGCTGCTGCGAG
Mouse-iNOS	ATGGCAACATCAGGTCGG	AACTGGGTGAACTCCAAGGT
Mouse-GAPDH	TGTGTCCGTCGTGGATCTGA	TTGCTGTTGAAGTCGCAGGAG

### Histology

The wound skin samples were harvested and fixed in 4% formaldehyde buffer. Skin tissues were dehydrated and subsequently paraffin-embedded. Skin tissue sections 5 μm thick were stained with haematoxylin and eosin (H&E) or Masson trichrome (MT) to visualise the histological structure and collagen deposition.

### Immunofluorescence

A 5 μm thick layer of paraffin-coated tissue was cut, decontaminated, and quenched with 3% H_2_O_2_ for 10 min. To reduce non-specific reactions, the sections were exposed to PBS containing 1% normal human serum, corresponding to the secondary antibody (Thermo Fisher Scientific, USA), and 2% BSA. To analyse M2 macrophages, staining with a monoclonal anti-CD206 and an anti-F4/80 was performed at 4 °C overnight, and then with a corresponding fluorescent secondary antibody. After washing, the slides were treated with DAPI (Sigma-Aldrich) for 5 min at room temperature to stain the nuclei, and the stained cells were examined under Leica Microsystems confocal microscope.

### Statistics

Each independent experiment included at least three replicates. The GraphPad Prism 8.0 software was used to conduct statistical tests. All data are presented as means ± standard error of the mean (SEM). Comparisons between two groups of samples were made using Student’s *t*-test, and comparisons between multiple groups of samples were made using one-way ANOVA. *p* < 0.05 being considered statistically significant (Mishra et al. 2019).

## Results

### The optimal concentration of RSV

**Figure 1.**
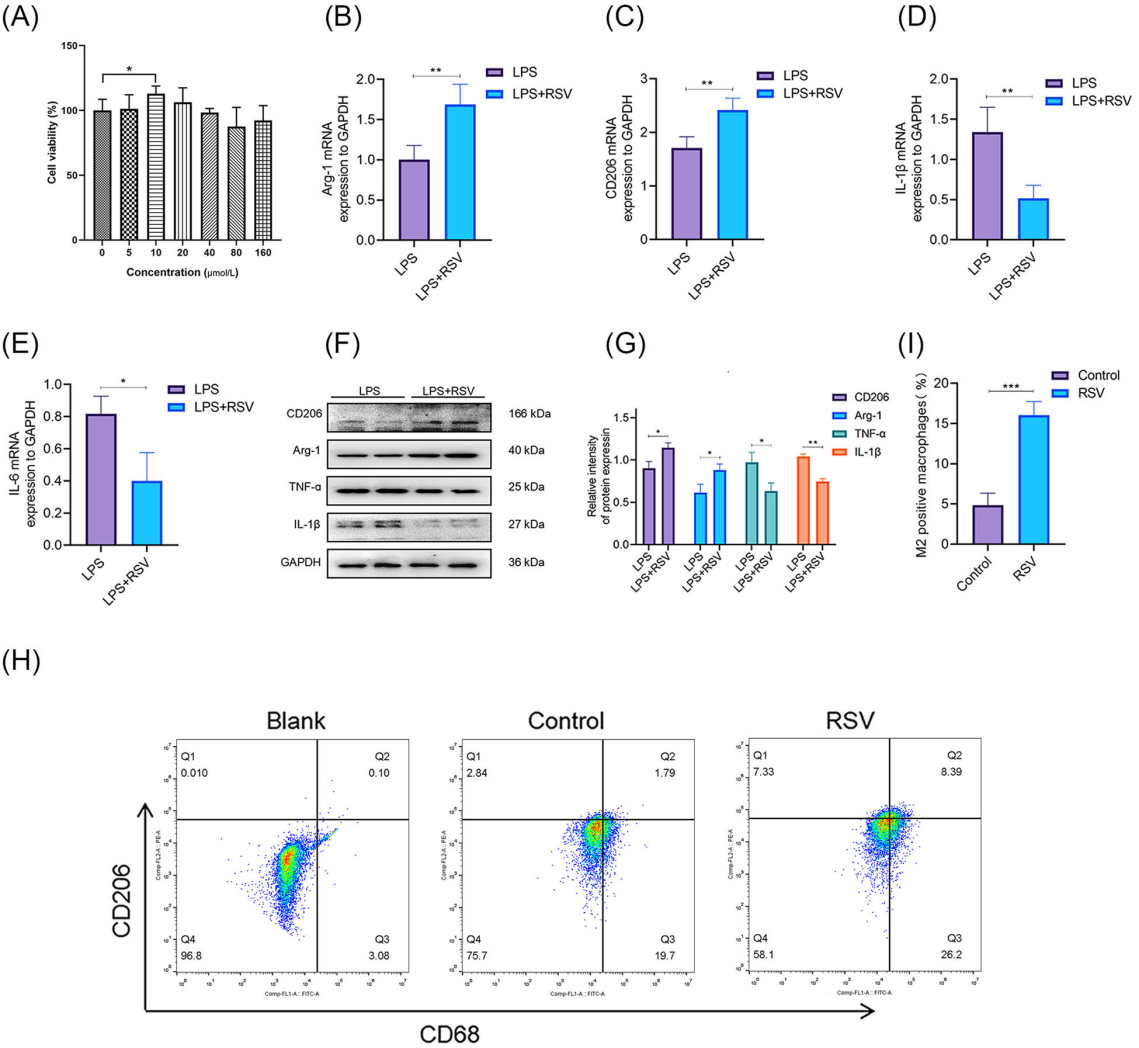
RSV‐induced M2 phenotype polarisation of macrophages *in vitro*. (A) Effect of different concentrations of RSV on cell viability. (B–E) mRNA expression of Arg-1, CD206, IL-1β, and IL-6 purified from the cells of the PBS group and RSV group were detected by RT-qPCR. (F,G) Western blot of CD206, Arg-1, IL-1β, and TNF-α and quantitative analysis of CD206, Arg-1, TNF-α, and IL-1β. GAPDH was used as an internal reference. (H) Representative flow cytometry was used to evaluate CD68 and surface markers CD206 in each group. (I) Quantification of the percentage of M2 macrophages. All results are presented as means ± SEMs; *n* = 3, *p < 0.05; **p < 0.01; ***p < 0.001.

To investigate the possible effects of different concentrations of RSV on THP-1 cells, a CCK-8 assay was performed. Excessive RSV concentrations significantly reduced the survival of THP-1 cells, the effective concentration of RSV for cell survival was 10 μmol/L (*p* < 0.05) without cytotoxicity to THP-1 cells (Figure 1(A)). This concentration will be used in subsequent experiments.

### RSV induces polarisation of M2 macrophages and reduces the secretion of pro-inflammatory factors *in vitro*

To evaluate the effects of RSV on the regulation of the inflammatory response and M2 macrophage polarisation under hyperglycaemia, THP-1 cells were treated with LPS in a high-glucose 1640 medium to simulate diabetes-induced chronic inflammation *in vitro*. The transcription and expression levels of M2-related genes (Arg-1 and CD206) and pro-inflammatory mediators (TNF-α, IL-6, and IL-1β) were detected using qPCR and Western blotting, respectively. The qPCR results showed that the use of RSV significantly increased the expression of Arg-1 and CD206 (*p* < 0.01) and decreased the expression of IL-1β and IL-6 (*p* < 0.05) compared with the LPS group (Figure 1(B–E)). WB results showed that the use of RSV significantly increased the expression of Arg-1 and CD206 (*p* < 0.05) and decreased the expression of TNF-α and IL-1β compared with the LPS group (*p* < 0.05) (Figure 1(F,G)). Flow cytometry results showed that the use of RSV significantly increased the percentage of M2 macrophages (CD206+) compared to the LPS group (*p* < 0.001) (Figure 1(H,I)). These results suggest that RSV promotes the polarisation of M2 macrophages *in vitro* and decreasing the secretion of pro-inflammatory factors TNF-α, IL-6 and IL-1β.

### Topical administration of RSV accelerates wound healing in diabetic mice

**Figure 2.**
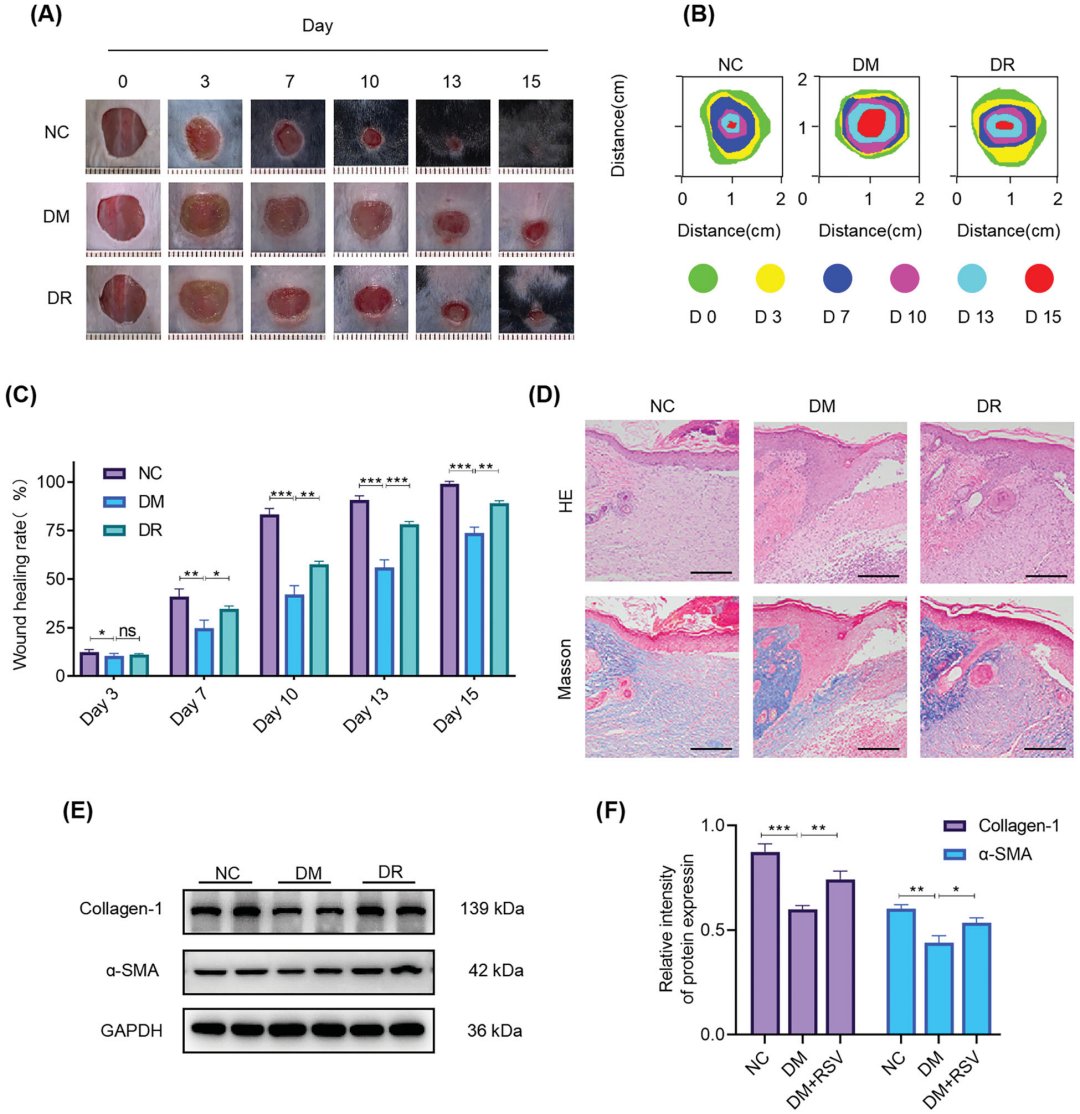
RSV expedites wound healing in diabetic mice. (A) Representative wound images on days 0, 3, 7, 10, 13, and 15 in different groups. Scale bar = 1 mm. (B) Simulation plots of the wound for 15 days for each group. (C) Quantification of wound closure rate at day 0–15 post-injury. (D) HE and Masson Trichrome staining of wounded skin tissue sections in different groups at day 10 post-injury. The scale bar is 50 μm. (E,F) Western blot of Collagen I and a-SMA and quantitative analysis of Collagen I and a-SMA at day 15 post-injury. GAPDH was used as an internal reference. All results are presented as means ± SEMs; *n* = 3, *p < 0.05; **p < 0.01; ***p < 0.001. NC: control group; DM: diabetes group; DR: diabetes + RSV group.

To explore the effect of RSV on diabetic wound healing, full-thickness wounds were generated on the backs of normal and STZ-induced diabetic mice. It was found that the wound healing process was significantly prolonged in the DM group and the wound healing rate was significantly lower than that in the NC group (*p* < 0.05). The use of RSV was able to accelerate the wound closure relative to the DM group (*p* < 0.05). The difference in wound healing was more pronounced from the seventh day (*p* < 0.01) and persisted throughout the healing process (Figure 2(A–C)). To further assess the healing quality, H&E and MT staining were performed to observe the histological structure and collagen deposition. The results showed that at day 15 the DM group displayed a thinner epidermis and less collagen deposition than normal wounds, and there was newly formed epithelial tissue in wounds treated with RSV. Besides, RSV treatment substantially promoted collagen deposition (Figure 2(D)). In addition, the expression of myofibroblast markers (α-SMA and Collagen I) in wound tissue was determined by western blotting on day 15 after injury. The expression of α-SMA and Collagen I was significantly downregulated in the DM group compared to the NC group (*p* < 0.01) and significantly increased after treatment with RSV (*p* < 0.05) (Figure 2(E,F)). These results suggest that RSV can promote wound healing in diabetic mice.

### RSV promotes M2 macrophage polarisation in diabetic wound

**Figure 3.**
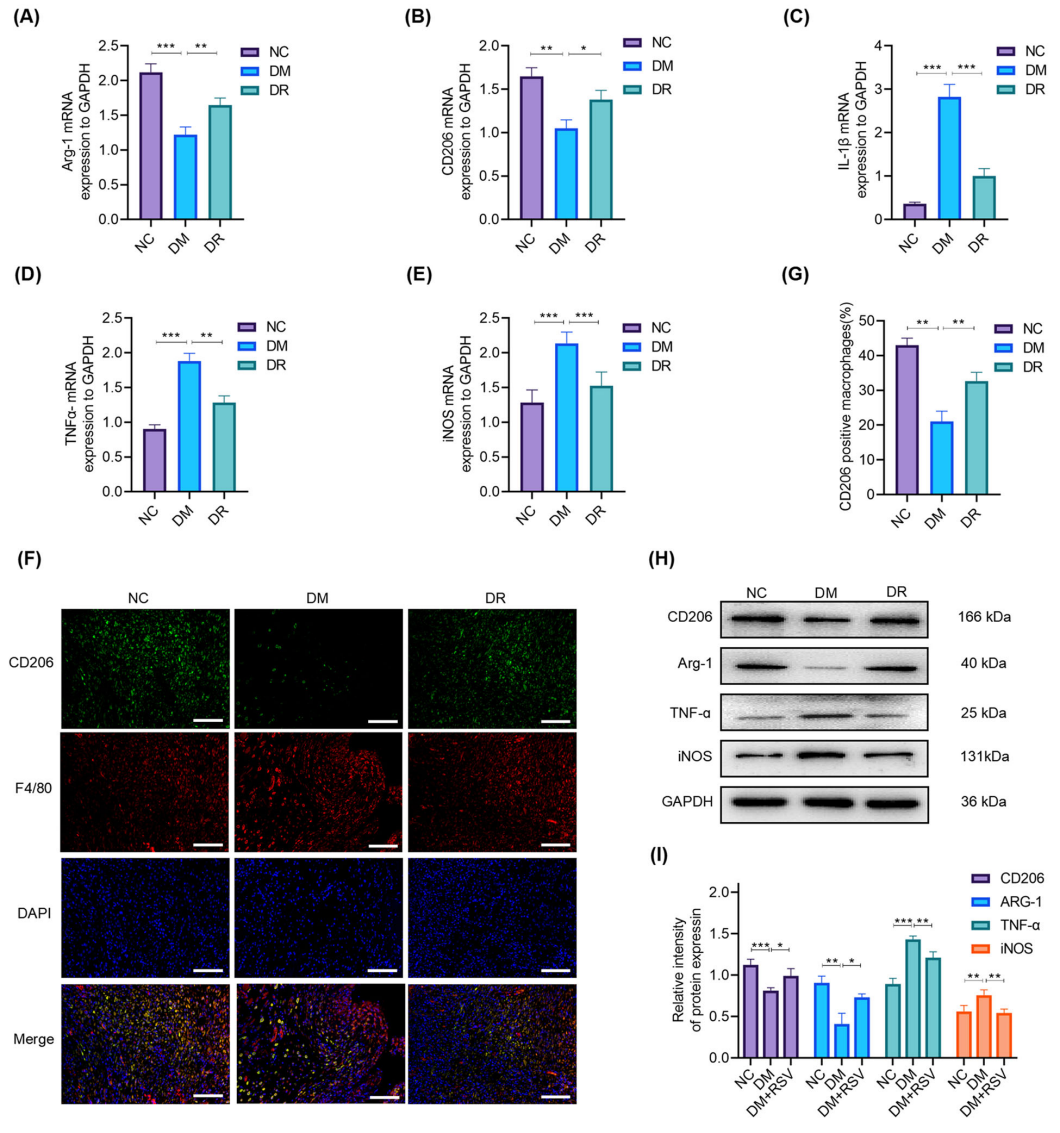
RSV regulates macrophage phenotype switch *in vivo*. (A–E) mRNA expression levels of CD206, Arg-1, iNOS, IL-1β, and TNF-α in wound tissues were determined by RT-qPCR. (F,G) Immunofluorescence staining showed the number of M2 macrophages in CN, DM, and DR groups. The scale bar is 50 µm. (H,I) Quantitative analysis of CD206, Arg-1, TNF-α, and iNOS at day 10 post-injury by western blot in the wound tissues of NC, DM, and DR groups. GAPDH was used as an internal reference. All results are presented as means ± SEMs; *n* = 3, *p < 0.05; **p < 0.01; ***p < 0.001. NC: control group; DM: diabetes group; DR: diabetes + RSV group.

To investigate whether local administration of RSV promotes the polarisation of M2 macrophages in diabetic wounds, wound-edge tissues were collected to detect the related indicators using RT-qPCR. Results revealed that M2 macrophage markers (Arg-1 and CD206) were significantly lower (*p* < 0.01) and pro-inflammatory markers (TNF-α, iNOS and IL-1β) were significantly higher (*p* < 0.001) in the DM group compared to the NC group. This trend in the DM group was attenuated by treatment with RSV (Figure 3(A–E)). Immunofluorescence results that the number of CD206+ cells were less in the DM group than in the NC group (*p* < 0.01), and treatment with RSV significantly increased the peri-wound CD206+ cell number (*p* < 0.01) (Figure 3(F,G)). These results were further confirmed by the WB, which showed significant differences between the different groups (*p* < 0.05) (Figure 3(H,I)). These results suggest that RSV promotes the polarisation of M2 macrophages in the wound tissue of diabetic mice.

### RSV promotes M2 macrophages polarisation and regulates inflammation via activating the PI3K/Akt signal

**Figure 4.**
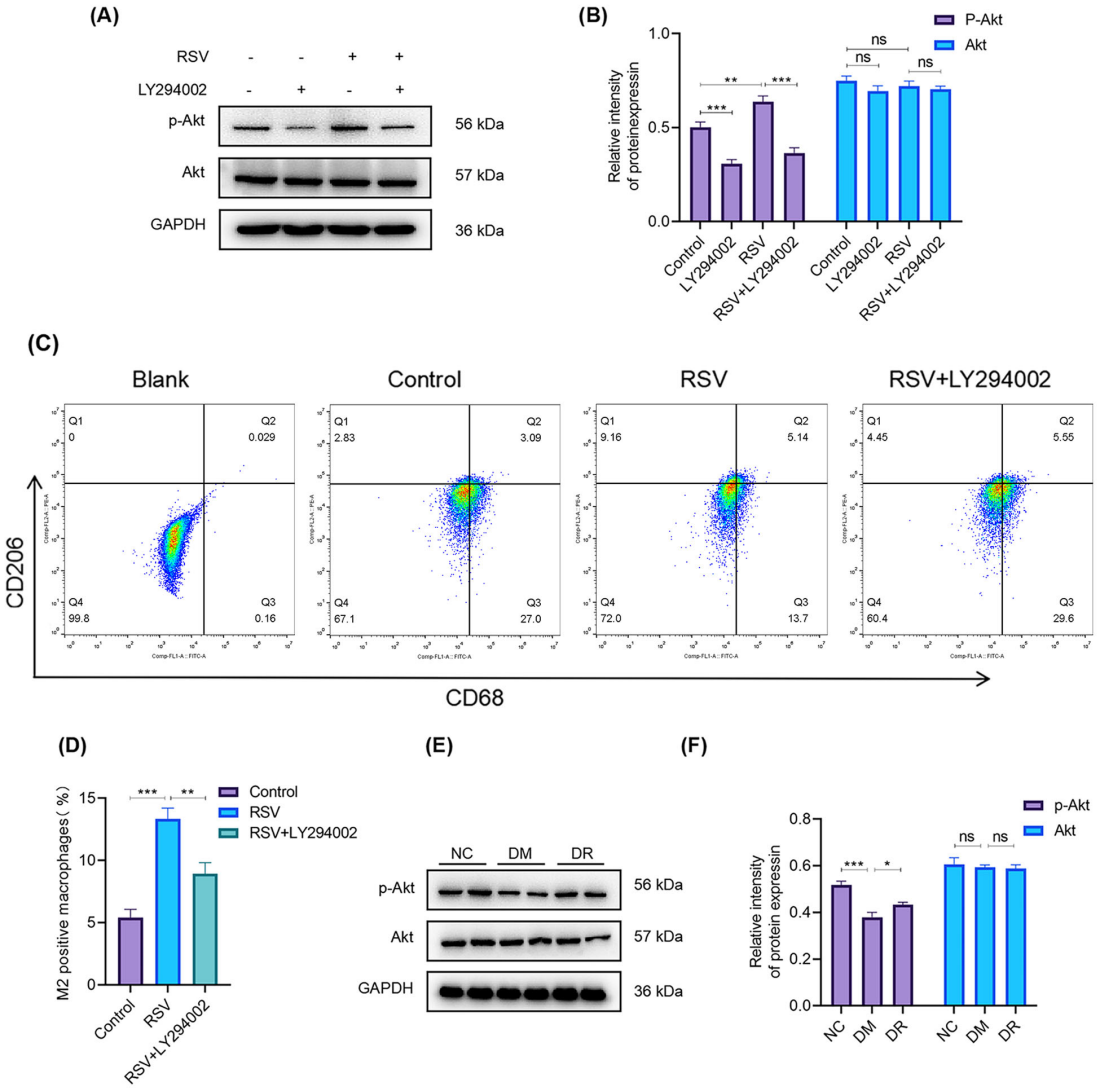
RSV promotes M2 macrophage polarisation and regulates inflammation *via* activating the PI3K-Akt signal. (A) Western blot analysis was performed to detect the protein expression of RSV in the PI3K/Akt signalling pathways. GAPDH was used as an internal reference. (B) Densitometry was used to quantify the Western blot bands shown in (A). (C) Representative flow cytometry analysis of the M2-like subset (CD206/CD68) proved that the M2 phenotype polarisation of macrophages stimulated by RSV was blocked by 25 µM LY294002. (D) The histogram depicted the quantification of the percentage of M2 macrophages. (E) Western blot analysis of the roles of RSV in PI3K/Akt pathways *in vivo*. GAPDH was used as an internal reference. (F) Densitometry was used to quantify the western blot bands shown in (E). All results are presented as means ± SEMs; *n* = 3, *p < 0.05; **p < 0.01; ***p < 0.001. NC: control group; DM: diabetes group; DR: diabetes + RSV group.^++^

PI3K/Akt signal transduction pathway is a crucial modulator of macrophage polarisation (Linton et al. 2019; Merecz-Sadowska et al. 2020). Therefore, we focussed on the relationship between RSV and PI3K/Akt signalling. To further determine the mechanism by which RSV induces M2 macrophage polarisation, cells were treated with PI3K pathway inhibitor (LY294002) for 24 h before RSV treatment. The results of WB showed that the level of p-Akt in THP-1-derived macrophages was significantly increased after RSV treatment (*p* < 0.01), and this effect was reversed after the use of LY294002 (*p* < 0.001) (Figure 4(A,B)). Flow cytometry results showed that the proportion of M2 macrophages in the RSV + LY294002 group was substantially lower than in the RSV group (*p* < 0.01) (Figure 4(C,D)). Besides, the phosphorylation levels of Akt in the wounds were also verified. p-Akt protein production was significantly lower in the DM group (*p* < 0.001), with the opposite effect produced by topical application of RSV (*p* < 0.05) (Figure 4(E,F)). These findings demonstrate that RSV promotes M2 macrophage polarisation in diabetic wounds by activating the PI3K/Akt signal transduction pathway.

## Discussion

DFU is one of the most common and severe complications of diabetes mellitus (Tuglo et al. 2022), and chronic inflammatory responses are the main reason for delayed or non-healing outcomes (Jeong et al. 2020). Our experimental results showed that RSV promoted wound healing in diabetic mice, and the polarisation of M2 macrophages played a key role in the treatment process. Topical application of RSV *in vivo* accelerated collagen deposition attenuated the inflammatory response of wound tissue with the decreased release of pro-inflammatory factors (IL-1β, iNOS, TNF-α), and promoted the polarisation of M2 macrophages (upregulation of Arg-1, CD206). Meanwhile, we conducted *in vitro* experiments, and the results were consistent with animal experiments in that RSV reduced the release of pro-inflammatory factors (IL-1β, IL-6, TNF-α) and induced M2 macrophage polarisation (upregulation of Arg-1, CD206). After the use of PI3K inhibitor LY294002, the therapeutic effect of RSV was reversed, the induction of M2 macrophages was attenuated, and the expression of p-Akt was decreased. The above experimental results suggest that RSV is able to induce M2 macrophage polarisation by the PI3K/Akt pathway and reduce the inflammatory response of wound tissue to exert therapeutic effects in promoting wound healing, which provides a new therapeutic idea to promote diabetic wound healing.

RSV is a natural non-flavonoid polyphenolic organic compound with positive regulatory effects on the immune response (Malaguarnera 2019), especially in alternative macrophage activation and the release of inflammatory mediators. The expression of proinflammation cytokines IL-1β, IL-6, TNF-α, and iNOS is inhibited by RSV in several diseases (Palsamy and Subramanian 2011; Zheng et al. 2013; Savi et al. 2016; Alrafas et al. 2019). In addition, many studies have suggested that RSV alleviates diabetes-related complications (Lee et al. 2011; Cao et al. 2019; Song et al. 2020). RSV has been reported to be a positive regulator of M2 macrophage polarisation in neuroinflammatory injury (Yang et al. 2017), myocardial infarction (Liu et al. 2019), and obesity-induced skeletal muscle inflammation (Shabani et al. 2020). Macrophage dysregulation plays an essential role in chronic inflammation in diabetic wounds (He et al. 2017); an unrestrained proinflammatory M1 macrophage population induced by diabetes is the main trigger for delayed or non-healing outcomes in diabetic wounds (Aitcheson et al. 2021). The promotion of M2 macrophage polarisation is an effective therapeutic strategy (Shen et al. 2020). Consistent with previous studies, we found that appropriate concentrations of RSV promote hyperglycaemic and LPS-stimulated macrophage polarisation and reduces the expression levels of pro-inflammatory factors.

Alternative macrophage polarisation is a critical link between inflammation and proliferation during cutaneous wound healing (Li et al. 2021). Transient M1 macrophages quickly switch to M2 macrophages during normal wound healing (Boniakowski et al. 2017). M1 macrophages produce pro-inflammatory cytokines and chemokines (IL-1β, IL-6, and TNF-α) to recruit appropriate cells and clear necrotic tissue, whereas M2 macrophages facilitate the opposite response, downregulating inflammation and contributing to wound repair (Hesketh et al. 2017; Kimball et al. 2018). However, macrophages fail to undergo timely alternative polarisation in diabetic wounds owing to persistent hyperglycaemia (Louiselle et al. 2021). Our experimental results are consistent with previous studies, diabetic wounds had fewer M2 macrophages and higher levels of pro-inflammatory cytokines and chemokines (IL-1β, TNF-α, and iNOS) than normal wounds. The abnormal inflammatory response was rescued by the topical application of RSV. It suggests that RSV may reduce the inflammatory response to wounds by inhibiting M1 macrophage polarisation, thereby reducing the release of pro-inflammatory factors. It is noteworthy that macrophage-derived cytokines induce fibroblast differentiation into myofibroblasts and regulate angiogenesis (Shook et al. 2018). *In vivo* experiments provided evidence that RSV-induced M2 macrophages affect the behaviour of other types of skin cells, increasing the expression of α-SMA and Collagen I in diabetic wounds. These outcomes concur with those of previous studies showing that RSV accelerates diabetic wound healing *via* neovascularization (Huang et al. 2019). The above results suggest that RSV can promote the formation of new blood vessels in diabetic mice wounds to accelerate the healing of diabetic wounds, and that both increased polarisation of M2 macrophages and reduced inflammatory response in this process.

Macrophages are key innate immune cells in the human body with high heterogeneity and plasticity. Macrophage activation is a tightly regulated phenomenon determined by several signalling cascades triggered by receptor stimuli or intracellular regulatory proteins (Vergadi et al. 2017). The different phases of macrophage activation are widely described as M1 and M2 polarisation, both consisting of a series of states that are dependent on convergent signals from inflammatory stimuli and the cellular environment (Murray et al. 2014). Under different microenvironments, macrophages can be polarised into the M1 type, activated by interferon-γ and LPS, and the M2 type, activated by interleukin-4, which plays a specific role in the innate immune response (Stein et al. 1992; Chow et al. 1999). Macrophage polarisation can be regulated by multiple pathways, including PI3K/Akt, AMPK, c-Myc, HIF, and PPARs signalling pathways (Wang et al. 2019). Previous studies have shown that RSV modulates inflammatory responses and macrophage polarisation *via* the PI3K/Akt signal transduction pathway (Merecz-Sadowska et al. 2020; Frontiers Production Office 2021), related to macrophage polarisation in diabetic wounds (Li et al. 2022). Akt activation is required for the induction of M2 macrophage polarisation because Akt inhibition eliminates the upregulation of M2 genes (Ruckerl et al. 2012; Byles et al. 2013; Covarrubias et al. 2015), which can promote M2 polarisation by activating the PI3K/Akt pathway. The PI3K/Akt pathway has been reported to be an important step in M2 macrophage polarisation, activation or overexpression of PI3K or AKT kinases can lead to decreased M1 macrophage polarisation, and intact PI3K/Akt signalling is an important factor driving the proliferation of M2 macrophage polarisation *in vivo* and *in vitro* (Luyendyk et al. 2008; Ruckerl et al. 2012; Covarrubias et al. 2015). Therefore, we investigated the effects of RSV on Akt phosphorylation. We found that the phosphorylation level of Akt was significantly increased in macrophages treated with RSV and that these effects were blocked by the PI3K inhibitor LY294002. These results indicate that RSV promoted M2 macrophage polarisation by activating the PI3K/Akt signal transduction pathway *in vitro*. Furthermore, p-Akt expression was upregulated following RSV administration in diabetic mice. Our findings suggest that RSV regulates macrophage polarisation *via* PI3K/Akt signal transduction in diabetic wounds. However, there are several limitations in our study. Although the article focuses on the potential mechanism of RSV on the polarisation of M2 macrophages, the research on M1 is relatively lacking. While the healing process of wound is complex and dynamic, the mechanism that deferred transformation from M1 to M2 macrophages still lack, and the research on the interaction of RSV on M1 and M2 is also worth further research. Thus, further in-depth research of RSV in the processes of the wound healing will provide us novel insights to clarify the therapeutic mechanisms of diabetic wound.

## Conclusions

This study is the first to investigate the mechanism of action of RSV in promoting diabetic wound healing using the PI3K/Akt pathway as an entry point. We demonstrated that RSV can induce M2 macrophage polarisation by activating the PI3K/Akt pathway, reduce inflammation to accelerate diabetic wound healing. The mechanism of action of RSV on promoting diabetic wound healing deserves further investigation.

## References

[CIT0001] Aitcheson SM, Frentiu FD, Hurn SE, Edwards K, Murray RZ. 2021. Skin wound healing: normal macrophage function and macrophage dysfunction in diabetic wounds. Molecules. 26(16):4917. 34443506 10.3390/molecules26164917 PMC8398285

[CIT0002] Alrafas HR, Busbee PB, Nagarkatti M, Nagarkatti PS. 2019. Resveratrol modulates the gut microbiota to prevent murine colitis development through induction of Tregs and suppression of Th17 cells. J Leukoc Biol. 106(2):467–480. 30897248 10.1002/JLB.3A1218-476RR PMC6863607

[CIT0003] Armstrong DG, Boulton AJM, Bus SA. 2017. Diabetic foot ulcers and their recurrence. N Engl J Med. 376(24):2367–2375. 28614678 10.1056/NEJMra1615439

[CIT0004] Bellary S, Kyrou I, Brown JE, Bailey CJ. 2021. Type 2 diabetes mellitus in older adults: clinical considerations and management. Nat Rev Endocrinol. 17(9):534–548. 34172940 10.1038/s41574-021-00512-2

[CIT0005] Boniakowski AE, Kimball AS, Jacobs BN, Kunkel SL, Gallagher KA. 2017. Macrophage-mediated inflammation in normal and diabetic wound Healing. J Immunol. 199(1):17–24. 28630109 10.4049/jimmunol.1700223

[CIT0006] Byles V, Covarrubias AJ, Ben-Sahra I, Lamming DW, Sabatini DM, Manning BD, Horng T. 2013. The TSC-mTOR pathway regulates macrophage polarization. Nat Commun. 4:2834. 24280772 10.1038/ncomms3834 PMC3876736

[CIT0007] Cao H, Ou J, Chen L, Zhang Y, Szkudelski T, Delmas D, Daglia M, Xiao J. 2019. Dietary polyphenols and type 2 diabetes: Human Study and Clinical Trial. Crit Rev Food Sci Nutr. 59(20):3371–3379. 29993262 10.1080/10408398.2018.1492900

[CIT0009] Chow JC, Young DW, Golenbock DT, Christ WJ, Gusovsky F. 1999. Toll-like receptor-4 mediates lipopolysaccharide-induced signal transduction. J Biol Chem. 274(16):10689–10692. 10196138 10.1074/jbc.274.16.10689

[CIT0010] Covarrubias AJ, Aksoylar HI, Horng T. 2015. Control of macrophage metabolism and activation by mTOR and Akt signaling. Semin Immunol. 27(4):286–296. 26360589 10.1016/j.smim.2015.08.001 PMC4682888

[CIT0011] Daemi A, Lotfi M, Farahpour MR, Oryan A, Ghayour SJ, Sonboli A. 2019. Topical application of *Cinnamomum* hydroethanolic extract improves wound healing by enhancing re-epithelialization and keratin biosynthesis in streptozotocin-induced diabetic mice. Pharm Biol. 57(1):799–806. 31760838 10.1080/13880209.2019.1687525 PMC6882457

[CIT0012] Dardmah F, Farahpour MR. 2021. *Quercus infectoria* gall extract aids wound healing in a streptozocin-induced diabetic mouse model. J Wound Care. 30(8):618–625. 34382850 10.12968/jowc.2021.30.8.618

[CIT0013] Eming SA, Krieg T, Davidson JM. 2007. Inflammation in wound repair: molecular and cellular mechanisms. J Invest Dermatol. 127(3):514–525. 17299434 10.1038/sj.jid.5700701

[CIT0014] Falanga V. 2005. Wound healing and its impairment in the diabetic foot. The Lancet. 366(9498):1736–1743. 10.1016/S0140-6736(05)67700-8 16291068

[CIT0016] Frontiers Production Office. 2021. Erratum: resveratrol alleviates dextran sulfate sodium-induced acute ulcerative colitis in mice by mediating PI3K/Akt/VEGFA pathway. Front Pharmacol. 12:797101. 34795595 10.3389/fphar.2021.797101 PMC8593133

[CIT0017] GHaraboghaz MNZ, Farahpour MR, Saghaie S. 2020. Topical co-administration of *Teucrium polium* hydroethanolic extract and *Aloe vera* gel triggered wound healing by accelerating cell proliferation in diabetic mouse model. Biomed Pharmacother. 127:110189. 32388242 10.1016/j.biopha.2020.110189

[CIT0018] He R, Yin H, Yuan B, Liu T, Luo L, Huang P, Dai L, Zeng K. 2017. IL-33 improves wound healing through enhanced M2 macrophage polarization in diabetic mice. Mol Immunol. 90:42–49. 28697404 10.1016/j.molimm.2017.06.249

[CIT0019] Hesketh M, Sahin KB, West ZE, Murray RZ. 2017. Macrophage phenotypes regulate scar formation and chronic wound healing. Int J Mol Sci. 18(7):1545. 28714933 10.3390/ijms18071545 PMC5536033

[CIT0020] Hu J, Zhang L, Liechty C, Zgheib C, Hodges MM, Liechty KW, Xu J. 2020. Long noncoding RNA GAS5 regulates macrophage polarization and diabetic wound healing. J Invest Dermatol. 140(8):1629–1638. 32004569 10.1016/j.jid.2019.12.030 PMC7384923

[CIT0021] Huang X, Sun J, Chen G, Niu C, Wang Y, Zhao C, Sun J, Huang H, Huang S, Liang Y, et al. 2019. Resveratrol promotes diabetic wound healing via SIRT1-FOXO1-c-Myc signaling pathway-mediated angiogenesis. Front Pharmacol. 10:421. 31068817 10.3389/fphar.2019.00421 PMC6491521

[CIT0022] Jeong S, Kim B, Park M, Ban E, Lee SH, Kim A. 2020. Improved diabetic wound healing by EGF encapsulation in gelatin-alginate coacervates. Pharmaceutics. 12(4):334. 32276508 10.3390/pharmaceutics12040334 PMC7238057

[CIT0023] Kaleci B, Koyuturk M. 2020. Efficacy of resveratrol in the wound healing process by reducing oxidative stress and promoting fibroblast cell proliferation and migration. Dermatol Ther. 33(6):e14357. 32996685 10.1111/dth.14357

[CIT0024] Kimball A, Schaller M, Joshi A, Davis FM, denDekker A, Boniakowski A, Bermick J, Obi A, Moore B, Henke PK, et al. 2018. Ly6C(Hi) blood monocyte/macrophage drive chronic inflammation and impair wound healing in diabetes mellitus. Arterioscler Thromb Vasc Biol. 38(5):1102–1114. 29496661 10.1161/ATVBAHA.118.310703 PMC5920725

[CIT0025] Komi DEA, Khomtchouk K, Santa Maria PL. 2020. A review of the contribution of mast cells in wound healing: involved molecular and cellular mechanisms. Clin Rev Allergy Immunol. 58(3):298–312. 30729428 10.1007/s12016-019-08729-w

[CIT0026] Lee SM, Yang H, Tartar DM, Gao B, Luo X, Ye SQ, Zaghouani H, Fang D. 2011. Prevention and treatment of diabetes with resveratrol in a non-obese mouse model of type 1 diabetes. Diabetologia. 54(5):1136–1146. 21340626 10.1007/s00125-011-2064-1 PMC4036531

[CIT0027] Li M, Hou Q, Zhong L, Zhao Y, Fu X. 2021. Macrophage related chronic inflammation in non-healing wounds. Front Immunol. 12:681710. 34220830 10.3389/fimmu.2021.681710 PMC8242337

[CIT0028] Li S, Ding X, Zhang H, Ding Y, Tan Q. 2022. IL-25 improves diabetic wound healing through stimulating M2 macrophage polarization and fibroblast activation. Int Immunopharmacol. 106:108605. 35149293 10.1016/j.intimp.2022.108605

[CIT0029] Li Y, Gao S, Shi S, Xiao D, Peng S, Gao Y, Zhu Y, Lin Y. 2021. Tetrahedral framework nucleic acid-based delivery of resveratrol alleviates insulin resistance: from innate to adaptive immunity. Nanomicro Lett. 13(1):86. 34138319 10.1007/s40820-021-00614-6 PMC8006527

[CIT0030] Linton MF, Moslehi JJ, Babaev VR. 2019. Akt signaling in macrophage polarization, survival, and atherosclerosis. IJMS. 20(11):2703. 31159424 10.3390/ijms20112703 PMC6600269

[CIT0031] Liu S, Du Y, Shi K, Yang Y, Yang Z. 2019. Resveratrol improves cardiac function by promoting M2-like polarization of macrophages in mice with myocardial infarction. Am J Transl Res. 11(8):5212–5226. 31497235 PMC6731431

[CIT0032] Louiselle AE, Niemiec SM, Zgheib C, Liechty KW. 2021. Macrophage polarization and diabetic wound healing. Transl Res. 236:109–116. 34089902 10.1016/j.trsl.2021.05.006

[CIT0033] Luyendyk JP, Schabbauer GA, Tencati M, Holscher T, Pawlinski R, Mackman N. 2008. Genetic analysis of the role of the PI3K-Akt pathway in lipopolysaccharide-induced cytokine and tissue factor gene expression in monocytes/macrophages. J Immunol. 180(6):4218–4226. 18322234 10.4049/jimmunol.180.6.4218 PMC2834303

[CIT0034] Malaguarnera L. 2019. Influence of resveratrol on the immune response. Nutrients. 11(5):946. 31035454 10.3390/nu11050946 PMC6566902

[CIT0035] Merecz-Sadowska A, Sitarek P, Śliwiński T, Zajdel R. 2020. Anti-inflammatory activity of extracts and pure compounds derived from plants via modulation of signaling pathways, especially PI3K/AKT in macrophages. IJMS. 21(24):9605. 33339446 10.3390/ijms21249605 PMC7766727

[CIT0036] Mishra P, Singh U, Pandey CM, Mishra P, Pandey G. 2019. Application of Student’s *t*-test, analysis of variance, and covariance. Ann Card Anaesth. 22(4):407–411. 31621677 10.4103/aca.ACA_94_19 PMC6813708

[CIT0037] Monteiro-Soares M, Ribeiro-Vaz I, Boyko EJ. 2019. Canagliflozin should be prescribed with caution to individuals with type 2 diabetes and high risk of amputation. Diabetologia. 62(6):900–904. 30941448 10.1007/s00125-019-4861-x

[CIT0038] Murray PJ, Allen JE, Biswas SK, Fisher EA, Gilroy DW, Goerdt S, Gordon S, Hamilton JA, Ivashkiv LB, Lawrence T, et al. 2014. Macrophage activation and polarization: nomenclature and experimental guidelines. Immunity. 41(1):14–20. 25035950 10.1016/j.immuni.2014.06.008 PMC4123412

[CIT0039] Palsamy P, Subramanian S. 2011. Resveratrol protects diabetic kidney by attenuating hyperglycemia-mediated oxidative stress and renal inflammatory cytokines via Nrf2-Keap1 signaling. Biochim Biophys Acta. 1812(7):719–731. 21439372 10.1016/j.bbadis.2011.03.008

[CIT0040] Patel S, Srivastava S, Singh MR, Singh D. 2019. Mechanistic insight into diabetic wounds: pathogenesis, molecular targets and treatment strategies to pace wound healing. Biomed Pharmacother. 112:108615. 30784919 10.1016/j.biopha.2019.108615

[CIT0041] Pignet AL, Schellnegger M, Hecker A, Kohlhauser M, Kotzbeck P, Kamolz LP. 2021. Resveratrol-induced signal transduction in wound healing. IJMS. 22(23):12614. 34884419 10.3390/ijms222312614 PMC8657598

[CIT0042] Radwan RR, Karam HM. 2020. Resveratrol attenuates intestinal injury in irradiated rats via PI3K/Akt/mTOR signaling pathway. Environ Toxicol. 35(2):223–230. 31633274 10.1002/tox.22859

[CIT0043] Ruckerl D, Jenkins SJ, Laqtom NN, Gallagher IJ, Sutherland TE, Duncan S, Buck AH, Allen JE. 2012. Induction of IL-4Ralpha-dependent microRNAs identifies PI3K/Akt signaling as essential for IL-4-driven murine macrophage proliferation *in vivo*. Blood. 120(11):2307–2316. 22855601 10.1182/blood-2012-02-408252 PMC3501641

[CIT0044] Saeedi P, Petersohn I, Salpea P, Malanda B, Karuranga S, Unwin N, Colagiuri S, Guariguata L, Motala AA, Ogurtsova K, et al. 2019. Global and regional diabetes prevalence estimates for 2019 and projections for 2030 and 2045: results from the International Diabetes Federation Diabetes Atlas, 9(th) edition. Diabetes Res Clin Pract. 157:107843. 31518657 10.1016/j.diabres.2019.107843

[CIT0045] Savi M, Bocchi L, Sala R, Frati C, Lagrasta C, Madeddu D, Falco A, Pollino S, Bresciani L, Miragoli M, et al. 2016. Parenchymal and stromal cells contribute to pro-inflammatory myocardial environment at early stages of diabetes: protective role of resveratrol. Nutrients. 8(11):729. 27854328 10.3390/nu8110729 PMC5133113

[CIT0046] Shabani M, Sadeghi A, Hosseini H, Teimouri M, Babaei Khorzoughi R, Pasalar P, Meshkani R. 2020. Resveratrol alleviates obesity-induced skeletal muscle inflammation via decreasing M1 macrophage polarization and increasing the regulatory T cell population. Sci Rep. 10(1):3791. 32123188 10.1038/s41598-020-60185-1 PMC7052230

[CIT0047] Shen T, Dai K, Yu Y, Wang J, Liu C. 2020. Sulfated chitosan rescues dysfunctional macrophages and accelerates wound healing in diabetic mice. Acta Biomater. 117:192–203. 33007486 10.1016/j.actbio.2020.09.035

[CIT0048] Shook BA, Wasko RR, Rivera-Gonzalez GC, Salazar-Gatzimas E, Lopez-Giraldez F, Dash BC, Munoz-Rojas AR, Aultman KD, Zwick RK, Lei V, et al. 2018. Myofibroblast proliferation and heterogeneity are supported by macrophages during skin repair. Science. 362(6417):909–909. 10.1126/science.aar2971 PMC6684198 30467144

[CIT0049] Song YJ, Zhong CB, Wu W. 2020. Resveratrol and diabetic cardiomyopathy: focusing on the protective signaling mechanisms. Oxid Med Cell Longev. 2020:7051845. 32256959 10.1155/2020/7051845 PMC7094200

[CIT0050] Stein M, Keshav S, Harris N, Gordon S. 1992. Interleukin 4 potently enhances murine macrophage mannose receptor activity: a marker of alternative immunologic macrophage activation. J Exp Med. 176(1):287–292. 1613462 10.1084/jem.176.1.287 PMC2119288

[CIT0051] Tuglo LS, Nyande FK, Agordoh PD, Nartey EB, Pan Z, Logosu L, Dei-Hlorlewu AE, Haligah DK, Osafo L, Taful S, et al. 2022. Knowledge and practice of diabetic foot care and the prevalence of diabetic foot ulcers among diabetic patients of selected hospitals in the Volta Region, Ghana. Int Wound J. 19(3):601–614. 34190402 10.1111/iwj.13656 PMC8874051

[CIT0052] Vergadi E, Ieronymaki E, Lyroni K, Vaporidi K, Tsatsanis C. 2017. Akt signaling pathway in macrophage activation and M1/M2 polarization. J Immunol. 198(3):1006–1014. 28115590 10.4049/jimmunol.1601515

[CIT0053] Wang L, Zhao H, Wang L, Tao Y, Du G, Guan W, Liu J, Brennan C, Ho CT, Li S. 2020. Effects of selected resveratrol analogues on activation and polarization of lipopolysaccharide-stimulated BV-2 microglial cells. J Agric Food Chem. 68(12):3750–3757. 32125844 10.1021/acs.jafc.0c00498

[CIT0054] Wang S, Liu R, Yu Q, Dong L, Bi Y, Liu G. 2019. Metabolic reprogramming of macrophages during infections and cancer. Cancer Lett. 452:14–22. 30905817 10.1016/j.canlet.2019.03.015

[CIT0055] Wolf SJ, Melvin WJ, Gallagher K. 2021. Macrophage-mediated inflammation in diabetic wound repair. Semin Cell Dev Biol. 119:111–118. 34183242 10.1016/j.semcdb.2021.06.013 PMC8985699

[CIT0056] Wynn TA, Vannella KM. 2016. Macrophages in tissue repair, regeneration, and fibrosis. Immunity. 44(3):450–462. 26982353 10.1016/j.immuni.2016.02.015 PMC4794754

[CIT0057] Yang L, Yang L, Tian W, Li J, Liu J, Zhu M, Zhang Y, Yang Y, Liu F, Zhang Q, et al. 2014. Resveratrol plays dual roles in pancreatic cancer cells. J Cancer Res Clin Oncol. 140(5):749–755. 24604347 10.1007/s00432-014-1624-4 PMC11823985

[CIT0058] Yang X, Xu S, Qian Y, Xiao Q. 2017. Resveratrol regulates microglia M1/M2 polarization via PGC-1alpha in conditions of neuroinflammatory injury. Brain Behav Immun. 64:162–172. 28268115 10.1016/j.bbi.2017.03.003

[CIT0060] Zheng X, Zhu S, Chang S, Cao Y, Dong J, Li J, Long R, Zhou Y. 2013. Protective effects of chronic resveratrol treatment on vascular inflammatory injury in streptozotocin-induced type 2 diabetic rats: role of NF-kappa B signaling. Eur J Pharmacol. 720(1–3):147–157. 24436987

[CIT0061] Zheng Y, Ley SH, Hu FB. 2018. Global aetiology and epidemiology of type 2 diabetes mellitus and its complications. Nat Rev Endocrinol. 14(2):88–98. 29219149 10.1038/nrendo.2017.151

